# *Streptomyces* Volatile Compounds Influence Exploration and Microbial Community Dynamics by Altering Iron Availability

**DOI:** 10.1128/mBio.00171-19

**Published:** 2019-03-05

**Authors:** Stephanie E. Jones, Christine A. Pham, Matthew P. Zambri, Joseph McKillip, Erin E. Carlson, Marie A. Elliot

**Affiliations:** a Department of Biology, McMaster University, Hamilton, Ontario, Canada; b Michael G. DeGroote Institute for Infectious Disease Research, McMaster University, Hamilton, Ontario, Canada; c Department of Chemistry, University of Minnesota, Minneapolis, Minnesota, USA; University of British Columbia

**Keywords:** *Streptomyces*, iron, microbial communities, pH, volatile

## Abstract

Bacteria and fungi produce a wide array of volatile organic compounds (VOCs), and these can act as chemical cues or as competitive tools. Recent work has shown that the VOC trimethylamine (TMA) can promote a new form of *Streptomyces* growth, termed “exploration.” Here, we report that TMA also serves to alter nutrient availability in the area surrounding exploring cultures: TMA dramatically increases the environmental pH and, in doing so, reduces iron availability. This, in turn, compromises the growth of other soil bacteria and fungi. In response to this low-iron environment, Streptomyces venezuelae secretes a suite of differentially modified siderophores and upregulates genes associated with siderophore uptake. Further reducing iron levels by limiting siderophore uptake or growing cultures in the presence of iron chelators enhanced exploration. Exploration was also increased when S. venezuelae was grown in association with the related low-iron- and TMA-tolerant *Amycolatopsis* bacteria, due to competition for available iron. We are only beginning to appreciate the role of VOCs in natural communities. This work reveals a new role for VOCs in modulating iron levels in the environment and implies a critical role for VOCs in modulating the behavior of microbes and the makeup of their communities. It further adds a new dimension to our understanding of the interspecies interactions that influence *Streptomyces* exploration and highlights the importance of iron in exploration modulation.

## Abstract (precis)

Microbial growth and community interactions are influenced by a multitude of factors. A new mode of *Streptomyces* growth—exploration—is promoted by interactions with the yeast Saccharomyces cerevisiae and requires the emission of trimethylamine (TMA), a pH-raising volatile compound. We show here that TMA emission also profoundly alters the environment around exploring cultures. It specifically reduces iron availability, and this in turn adversely affects the viability of surrounding microbes. Paradoxically, *Streptomyces* bacteria thrive in these iron-depleted niches, both rewiring their gene expression and metabolism to facilitate iron uptake and increasing their exploration rate. Growth in close proximity to other microbes adept at iron uptake also enhances exploration. Collectively, the data from this work reveal a new role for bacterial volatile compounds in modulating nutrient availability and microbial community behavior. The results further expand the repertoire of interspecies interactions and nutrient cues that impact *Streptomyces* exploration and provide new mechanistic insight into this unique mode of bacterial growth.

## INTRODUCTION

Bacteria and fungi frequently live in densely populated multispecies communities. These microbes produce a vast array of molecules capable of modulating community dynamics, including specialized metabolites and volatile organic compounds (VOCs) (1). Soil environments are particularly complex: not only are they home to multitudes of microbes, but they are also heterogeneous systems, containing solid microenvironments and nutrient gradients connected by networks of water- and air-filled pores (2). To date, the majority of studies on interspecies competition in microbial communities have focused on the effects of specialized metabolites. These compounds effectively mediate microbial communication and competition, but their effects are limited to nearby interactions, due to their limited diffusion capabilities. In contrast, VOCs are low-molecular-weight compounds that can rapidly diffuse across water channels and air pockets and consequently can act as longer-range signals (3). The biological roles of VOCs are only now starting to be dissected, and initial studies are showing that they can have broad effects on both their producing organisms and their neighbors. Indeed, VOCs can alter the antibiotic resistance profiles of bacteria, act as antifungal or antibiotic compounds, promote group behaviors such as motility and biofilm formation, and induce widespread changes in the gene expression of nearby microbes (4).

One group of prolific volatile producers is composed of the *Streptomyces* bacteria (5–7). In soil, these bacteria are best known for their ability to produce a vast array of specialized metabolites and for their complex, filamentous life cycle (8, 9). Recent work has, however, revealed that *Streptomyces* species also use volatile compounds to promote an alternative growth strategy known as “exploration” (10, 11). In the model species Streptomyces venezuelae, exploration is initiated in response to the production of the VOC trimethylamine (TMA), which promotes the rapid spreading of explorer cells across surfaces. TMA production dramatically alters the surrounding environment, raising the pH to levels approaching 9.5, and further serves as a *Streptomyces* communication cue, inducing exploration in physically separated streptomycete colonies. TMA-mediated induction of exploration appears to be a function of its alkalinity, as other alkaline VOCs (e.g., ammonia) can induce exploration in a similar manner. TMA is also effective as a weapon against nonstreptomycetes: both exploring *Streptomyces* colonies and TMA solutions reduce the survival of other soil bacteria, including Bacillus subtilis and Micrococcus luteus (11).

The results seen with TMA with respect to environmental alkalinization, *Streptomyces* exploration, and the growth of other microbes suggest far-reaching effects for this VOC. How TMA affects microbial community dynamics and impacts the growth of other microbes is, however, not clear. Here, we demonstrated that TMA emitted by *Streptomyces* explorer cells reduces the survival of other soil bacteria and fungi by starving them of iron—a nutrient that is critical for microbial growth and viability and whose bioavailability is inversely correlated with environmental pH. Our results suggest that *Streptomyces* thrive within those high-pH, self-induced iron-depleted environments by secreting siderophores and rewiring gene expression to maximize siderophore uptake. We show that iron depletion by other microbes, or by iron chelators, can enhance *Streptomyces* exploration, suggesting that low iron is a driver of exploratory growth. Taken together, our results reveal a new way in which *Streptomyces* can alter the availability of environmental iron and can in turn influence their own growth and behavior and that of other members of the surrounding microbial communities. Our findings further suggest that iron depletion has the potential to activate a positive-feedback loop that promotes the continued expansion of exploring cultures.

## RESULTS

### Environmental iron availability impacts the survival of bacteria and fungi.

Iron in the environment exists predominantly in its poorly soluble ferric form (Fe^3+^). To facilitate iron acquisition, bacteria release iron-chelating siderophores (12). These small molecules bind ferric iron, and the resulting siderophore-iron complexes are taken back up into cells through dedicated membrane transporters, after which iron is released in its ferrous form (Fe^2+^). In alkaline environments, iron solubility drops by ∼1,000-fold with each unit rise in pH, due to ferric iron forming stable complexes with hydroxide ions. This further lowers iron solubility and reduces iron binding by siderophores (13–15). S. venezuelae exploration requires an alkaline environment, which S. venezuelae creates by releasing the volatile compound TMA. TMA emission also results in dramatically decreased survival of other soil-dwelling bacteria (11). Consequently, we wondered whether low levels of iron availability could explain the reduced survival observed for other microbes exposed to exploration-associated VOCs.

**FIG 1:**
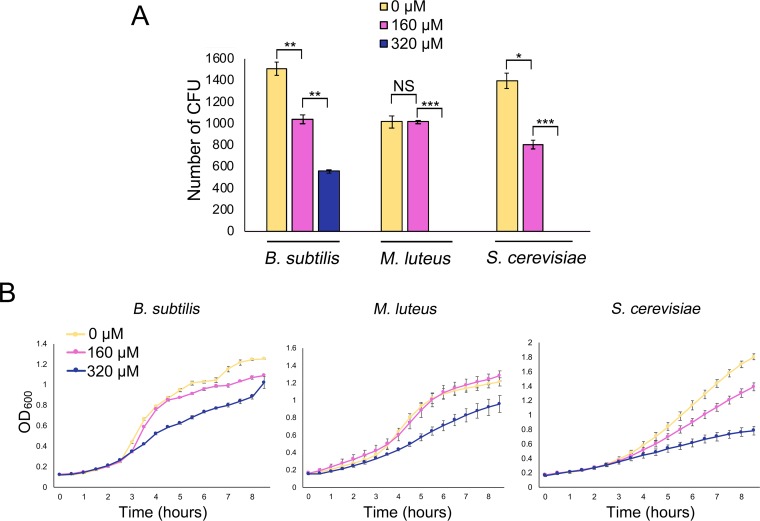
Iron availability impacts the growth and survival of bacteria and fungi. (A) Quantification of colony forming units (CFU) for B. subtilis and M. luteus on LB agar medium and S. cerevisiae on YPD agar medium, supplemented with 0, 160, or 320 µM 2,2'-dipyridyl. Plates were incubated for 48 h. All values represent means ± standard errors of results from three or four biological replicates. Asterisks indicate statistically significant differences (*, *P* values of 0.05 to 0.01; **, *P* values of 0.01 to 0.005; ***, *P* values below 0.005; NS, no significant difference) as determined by a Student's *t* test. Note that M. luteus and S. cerevisiae did not grow at 320 µM 2,2'-dipyridyl. (B) Growth curves of B. subtilis and M. luteus in liquid LB medium and S. cerevisiae in liquid YPD medium, supplemented with 0, 160, or 320 µM 2,2'-dipyridyl and grown for 8 h. Values represent means ± standard errors of results from three biological replicates.

To address this possibility, we first tested the extent to which low iron affected the growth of the soil bacteria B. subtilis and M. luteus, as well as the fungus Saccharomyces cerevisiae. We compared the growth levels of these strains on solid medium relative to their growth on medium supplemented with the iron-specific chelator 2,2'-dipyridyl (here referred to as dipyridyl) (160 or 320 µM). We observed a linear growth decrease for B. subtilis on LB medium containing dipyridyl compared with LB medium alone: colony numbers were reduced by ∼30% on 160 µM dipyridyl, and they dropped a further 30% on 320 µM dipyridyl (Fig. 1A). In contrast, for M. luteus, colony numbers were equivalent on LB without dipyridyl and on LB with 160 µM dipyridyl, although the colonies were smaller under conditions of growth on dipyridyl, suggesting that the low availability of iron slowed the growth of these organisms. With 320 µM dipyridyl, however, no M. luteus colonies survived (Fig. 1A). Finally, in the case of S. cerevisiae, colony numbers decreased by an average of ∼40% on yeast-peptone-dextrose (YPD) medium with 160 µM dipyridyl; colonies failed to grow altogether on plates containing 320 µM dipyridyl (Fig. 1A).

We also tested the effect of dipyridyl on the liquid culture growth of each of these organisms. Each microbe was grown in liquid medium (LB for B. subtilis and M. luteus; YPD for S. cerevisiae), and the growth levels were compared with that in medium supplemented with 160 µM or 320 µM dipyridyl. As we saw for the solid-grown cultures, the growth rate for each strain decreased as dipyridyl concentrations increased (Fig. 1B). We found that supplementing these cultures with iron could restore wild-type growth levels to each microbe (see Fig. S1 in the supplemental material), indicating that the growth inhibition was iron specific and was unrelated to other potential toxic effects of dipyridyl. Collectively, these experiments verified that iron was important for the growth of these microorganisms.

10.1128/mBio.00171-19.1 FIG S1 Effects of dipyridyl, manganese, and copper on microbial growth. (A) Growth curves of B. subtilis and M. luteus in liquid LB medium and S. cerevisiae in liquid YPD medium supplemented with 0, 160, or 320 µM dipyridyl or dipyridyl with 2 mM iron and grown for 8 h. Values represent means ± standard errors of results from three biological replicates. (B) Percent survival of B. subtilis (top), M. luteus (middle), and S. cerevisiae (bottom) cells grown adjacent to wells containing water or TMA (5%), on media alone, or media supplemented with 1 mM FeCl_3_, MnCl_2_, or CuCl_2_. Values represent means ± standard errors of results from three independent replicates. Download FIG S1, TIF file, 5.1 MB. Copyright © 2019 Jones et al. 2019 Jones et al. This content is distributed under the terms of the Creative Commons Attribution 4.0 International license.

### Iron supplementation rescues microbial growth in the presence of explorer cells.

**FIG 2:**
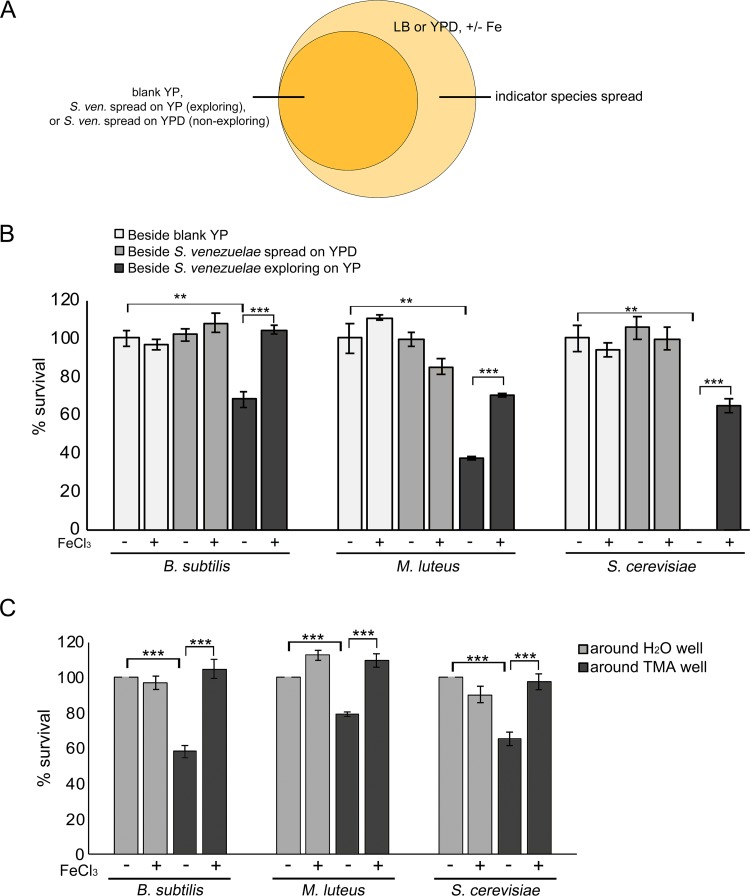
Iron supplementation restores the growth of microbes exposed to VOCs. (A) Schematic of the experimental set-up. Plates comprising uninoculated (blank) YP agar, S. venezuelae (*S. ven*) exploring on YP agar, or nonexploring S. venezuelae on YPD medium were incubated in smaller dishes for 10 days. After 10 days, an indicator strain (B. subtilis, M. luteus, or S. cerevisiae) was spread on medium (with or without 1 mM FeCl3 supplementation) in the outside dish. (B) Quantification of B. subtilis, M. luteus, or S. cerevisiae growth/survival on medium with or without FeCl_3_ supplementation, following growth adjacent to (beside) exploring S. venezuelae, nonexploring S. venezuelae, or uninoculated medium. (C) Experiments were conducted as described for panels A and B, only with H_2_O or TMA solutions replacing S. venezuelae-inoculated YP/YPD media. Plates were incubated at room temperature for 2 days. B. subtilis, M. luteus, or S. cerevisiae growth/survival was quantified following incubation on medium with or without FeCl_3_ supplementation, adjacent to (around) wells with H_2_O or TMA solutions. Values in panels B and C represent means ± standard errors of results from three or four replicates, and statistical significance was determined using one-way analysis of variance (ANOVA), followed by Tukey’s multiple-comparison test. Asterisks (*) indicate *P* values (**, *P* value of 0.01 to 0.005; ***, *P* value below 0.005).

Having demonstrated that iron sufficiency was essential for robust growth by B. subtilis, M. luteus, and S. cerevisiae, we sought to test our hypothesis that the volatile compounds produced by exploring S. venezuelae reduced the survival of other microbes by creating an alkaline, iron-deficient environment. We set up a small petri dish that had YPD agar (nonexploring medium) or YP agar (exploring medium) inside but that was physically separated from a larger dish containing either medium alone or medium supplemented with additional iron. The small petri plates were inoculated with S. venezuelae and incubated for 10 days, after which B. subtilis, M. luteus, or S. cerevisiae cells were spread on the larger, surrounding agar plates (Fig. 2A). Growth of each indicator microbe was assessed after 2 days. When the microbes were grown adjacent to YP plates without *Streptomyces* inoculum or to nonexploring S. venezuelae cultures on YPD medium, the colony numbers for each microbe were similar on all plates, irrespective of the presence or absence of iron supplementation (Fig. 2B). As the presence of extra iron did not enhance growth, the results suggested that iron was unlikely to be limiting for the growth of these organisms under these conditions.

In contrast, when these microbes were grown next to exploring S. venezuelae on YP medium, the colony numbers on the surrounding agar plates differed drastically depending on their iron supplementation status. Growing B. subtilis, M. luteus, and S. cerevisiae adjacent to exploring S. venezuelae on medium without added iron led to average reductions in colony numbers of 32%, 63%, and 100%, respectively, relative to controls (those grown on plates adjacent to blank YP or nonexploring S. venezuelae on YPD) (Fig. 2B). In each case, growth was partially (M. luteus and S. cerevisiae) or fully (B. subtilis) restored by iron supplementation (Fig. 2B). These data suggest that the alkaline environments created by exploring S. venezuelae reduced the viability of other soil microbes, at least in part by starving them of iron.

To determine whether this was a TMA-dependent phenomenon or whether it was due to other volatiles produced by exploring *Streptomyces*, we set up equivalent assays where S. venezuelae colonies were replaced with water or TMA (11) (Fig. 2C). Around these water/TMA-containing wells were spread the three indicator organisms on agar medium, with or without iron supplementation. We quantified their growth after 2 days. For each microbe, the addition of iron had little impact on cell survival when cultures were grown next to water-containing wells: B. subtilis growth was unaffected, while there was a slight increase in growth observed for M. luteus and a slight decrease for S. cerevisiae (Fig. 2C).

In contrast, iron supplementation had a significant effect on survival and growth when cells were plated adjacent to TMA-containing wells. Exposure of B. subtilis, M. luteus, and S. cerevisiae to TMA during growth on plates without iron supplementation led to reductions in the growth/survival of these strains of 42%, 21%, and 35%, respectively (on average), compared with those grown on plates adjacent to water wells (Fig. 2C). As seen for the exploring culture experiments described above, iron supplementation restored the growth of TMA-exposed cultures to levels equivalent to those seen around the water wells (Fig. 2C). This indicated that TMA emission by exploring S. venezuelae functioned to inhibit the growth of other microbes by limiting iron availability. It appeared, however, either that exploring S. venezuelae produced more TMA than we were using in these assays or that additional volatile compounds influenced the growth of the yeast S. cerevisiae and, to a lesser extent, M. luteus, as the growth of these organisms was more strongly impacted by exploring S. venezuelae than by TMA exposure.

To confirm that the effect that we were observing was specific for iron, we tested the impact of supplementation of media with manganese and copper—two other divalent cations whose solubility is also affected by alkaline conditions (14, 15). We found that neither of these cations rescued the growth of B. subtilis or M. luteus (Fig. S1). For S. cerevisiae, manganese supplementation was detrimental to growth, and manganese addition in the presence of TMA appeared to be highly toxic. In contrast, addition of copper enhanced growth by ∼15% under conditions of exposure to both water and TMA, unlike the results seen with iron, which specifically enhanced growth upon exposure to TMA (Fig. S1). Taken together, these results suggest that the growth defects observed in the presence of TMA were largely a result of iron starvation.

### Desferrioxamines are produced during exploration.

**FIG 3:**
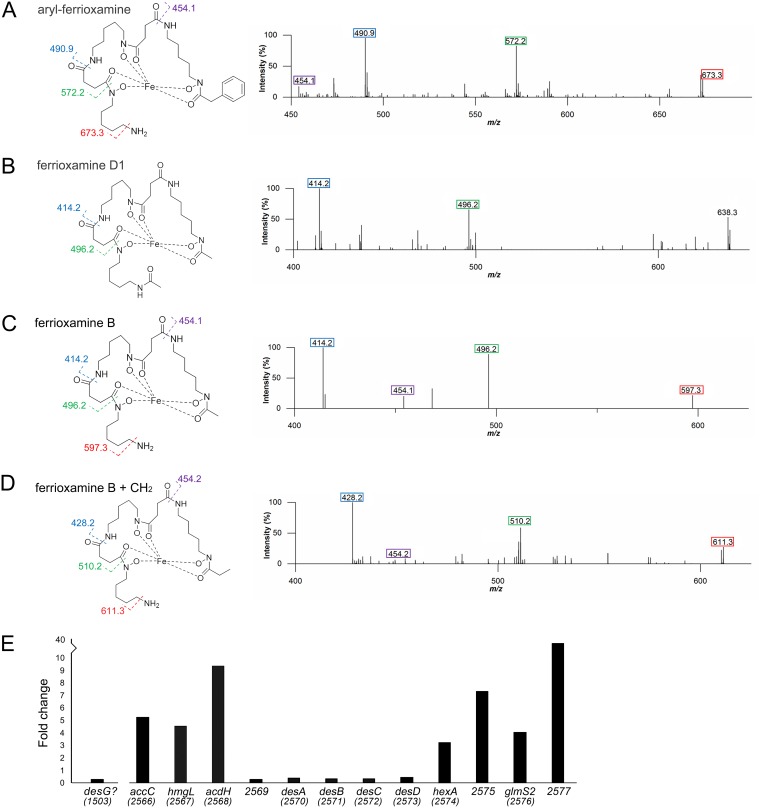
Explorer cells produce ferrioxamines. Panels A to D show accurate mass fragmentation (MS^2^) data and molecular structures of associated ferrioxamine (iron-complexed desferrioxamine) compounds. Fragments that are common to this class of molecules have been indicated with colored boxes on each molecule, along with colored numbers above the associated fragment in the spectra. (A) Aryl-ferrioxamine, from S. venezuelae exploring on YP and S. venezuelae exploring beside S. cerevisiae on YPD. (B) Ferrioxamine D1, from S. venezuelae exploring on YP and S. venezuelae exploring beside S. cerevisiae on YPD. (C) Ferrioxamine B, from S. venezuelae exploring on YP and S. venezuelae exploring beside S. cerevisiae on YPD. (D) Ferrioxamine B plus CH_2_ (where the CH_2_ is depicted as an ethyl group, as the methylene addition is on the right-hand side of the molecule and is most likely located in the acyl tail) from S. venezuelae exploring beside S. cerevisiae on YPD. (E) Normalized transcript levels for *desG* and genes in the S. venezuelae desferrioxamine biosynthetic cluster (as defined by antiSMASH [20]) in explorer cells, divided by those determined for nonexploring cells. The associated gene names or *sven* gene numbers are shown below each gene.

As iron supplementation was able to restore the growth of other microbes in the presence of TMA/*Streptomyces* volatile compounds, this suggested that these volatile compounds were responsible for creating a low-iron environment. This then raised the issue of how exploring S. venezuelae dealt with these low-iron conditions. To begin addressing this point, we examined the metabolic output of exploring cultures. Using liquid chromatography coupled with mass spectrometry (LC-MS), we compared the metabolites produced by exploring cultures (for S. venezuelae grown alone on YP medium and directly beside S. cerevisiae on YPD medium—two conditions that promote S. venezuelae exploration) versus nonexploring colonies (S. venezuelae grown alone on YPD or MYM [maltose, yeast extract, malt extract] medium) (Fig. 3A to D). We found that all exploring cultures produced analogs of the ferrioxamine (iron-complexed desferrioxamine) siderophore, including ferrioxamine B and D and an aryl-functionalized ferrioxamine (Fig. 3A to C). Exploring colonies grown in association with S. cerevisiae on YPD also produced the unusual ferrioxamine B+CH_2_, reported previously by Cruz-Morales et al. (16) (Fig. 3D). Importantly, (des)ferrioxamines were not detected in nonexploring cultures (Fig. S2) and were not produced by S. cerevisiae (Fig. S2), suggesting that the production of these molecules was specific to exploring S. venezuelae cultures under these growth conditions.

10.1128/mBio.00171-19.2 FIG S2 Ferrioxamine B production is observed only for exploring S. venezuelae. Extracted ion chromatogram for ferrioxamine B (*m*/*z* 614) from YPD medium alone (pink), S. cerevisiae grown alone on YPD (yeast; blue), S. venezuelae grown alone on YPD (no exploration/stationary cells; red), and S. venezuelae cultured with yeast (explorer cells; black). Download FIG S2, TIF file, 0.5 MB. Copyright © 2019 Jones et al. 2019 Jones et al. This content is distributed under the terms of the Creative Commons Attribution 4.0 International license.

In parallel, we revisited our previously generated transcriptome sequencing (RNA-seq) data (11) to determine whether exploring cultures were generally exhibiting a transcriptional profile consistent with iron starvation. While the regulation of iron homeostasis has not been studied in S. venezuelae, investigations in the closely related species Streptomyces coelicolor have revealed that the desferrioxamine biosynthetic gene cluster (*desABCD*) is controlled by the iron-responsive regulator DesR/DmdR (17). DesR/DmdR binds to a site overlapping the −10 promoter region upstream of *desA*, repressing the expression of the *des* genes when iron is abundant. Under iron-deficient conditions, transcription repression is alleviated, and transcription of the *des* operon increases (17). A similar situation appears to exists for S. venezuelae, which encodes a DesR/DmdR homologue (S. venezuelae 4209 [SVEN_4209]) sharing 92% sequence identity (96% sequence similarity) with the S. coelicolor protein and which has a similar “iron box” overlapping the promoter of the *des* operon (Fig. S3).

10.1128/mBio.00171-19.3 FIG S3 Organization of the desferrioxamine biosynthetic cluster and its control by DesR/DmdR. (A) The desferrioxamine biosynthetic cluster, with gene names and associated *sven* numbers shown below each gene. Promoter locations are shown as elevated, thin arrows. The promoter region upstream of *desA* is indicated with a box and a line coming down from it in the cluster diagram, and the sequence is shown below with the predicted DesR/DmdR binding site highlighted in gray. (B) Alignment of the promoter regions for *desA* in S. coelicolor (top) and S. venezuelae (bottom). (C) Alignment of the DesR/DmdR regulators from S. coelicolor (SCO4394) and S. venezuelae (SVEN_4209). Download FIG S3, TIF file, 1.8 MB. Copyright © 2019 Jones et al. 2019 Jones et al. This content is distributed under the terms of the Creative Commons Attribution 4.0 International license.

Unexpectedly, we found that *desABCD* (*sven_2570–73*) transcript levels were not upregulated in exploring cultures relative to static cultures (Fig. 3E), although transcript levels for the genes flanking the *des* operon were significantly increased under exploring conditions (Fig. 3E); it is not clear what role these gene products play in desferrioxamine synthesis. Work by Cruz-Morales and colleagues had suggested that an enzyme dubbed DesG was responsible for generating aryl-desferrioxamines (16). Searches for equivalent proteins in S. venezuelae revealed the best candidate to be SVEN_1503. Its coding sequence was also preceded by an iron box (TTAGGTCAGCCTAA, beginning 57 nucleotides upstream of the start codon), but, like *desABCD*, its transcript levels were not upregulated during exploration (Fig. 3E).

### Oligopeptide transporters influence exploration and culture response to iron.

**FIG 4:**
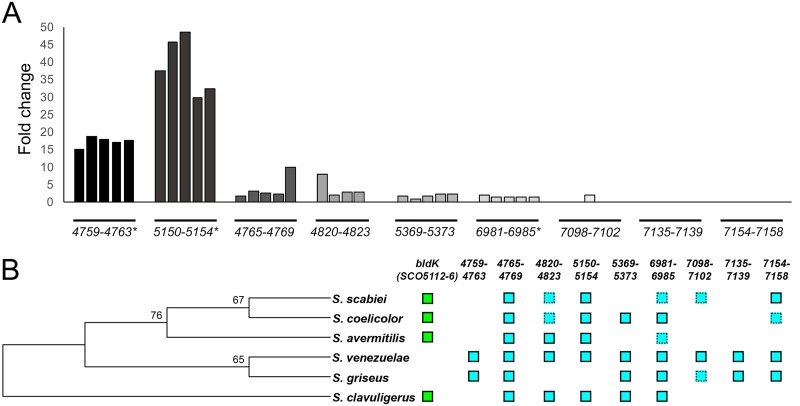
Upregulation of two gene clusters associated with siderophore transport in explorer cells. (A) Normalized transcript levels for S. venezuelae clusters homologous to the S. coelicolor *bldKABCDE* locus; values representing normalized transcript levels in S. venezuelae explorer cells were divided by values representing those in nonexploring cells. The associated *sven* gene numbers are shown below each cluster, and asterisks beside gene numbers indicate that there are genes in the cluster that are significantly differentially expressed in exploring versus nonexploring cultures, based on calculated false-discovery-rate (*q*) values. (B) Phylogenetic distribution of *bldK-*like gene clusters in well-studied *Streptomyces* species. The *Streptomyces* phylogeny was created using aligned 16S rRNA sequences, and a maximum likelihood tree was built using MEGA, with 100 bootstrap replicates to infer support values of nodes. Green boxes, presence of reciprocally orthologous *bldK* clusters; turquoise boxes, presence of reciprocally orthologous *bldK-*like clusters from S. venezuelae; hatched turquoise boxes, presence of orthologues to a subset of genes from each cluster.

Upon further analysis of our RNA-seq data, we observed that two of the most highly upregulated gene clusters in exploring cultures (*sven_5150–53* and *sven_4759–63*) encoded ATP-binding cassette (ABC) transporter systems (Fig. 4A). The characterized homologues closest to these genes in the model S. coelicolor system were in the *bldK* locus (*SCO5112–16*). Recent work has suggested that BldK transporters may function in ferrioxamine siderophore uptake (18). Increased expression of these two *bldK-*like transporter gene clusters in S. venezuelae implied that exploring cultures may adapt to alkaline, low-iron environments by coupling increased desferrioxamine production with enhanced ferrioxamine uptake.

We searched for other genes in S. venezuelae that were homologous to the S. coelicolor *bldK* locus and found that S. venezuelae harbored seven additional *bldK-*like clusters, many of which had orthologues in other well-studied streptomycetes (Fig. 4B). Of these, three (*sven_7098–02*, *sven_7135–39*, and *sven_7154–58*) were not expressed in exploring or static cultures, three (*sven_4765–69*, *sven_4820–23*, and *sven_5369–73*) were expressed at low levels in both exploring and static cultures, and one (*sven_6981–85*) was expressed at intermediate levels in exploring cultures but was upregulated only modestly (∼1.7-fold) in exploring versus static cultures and is predicted to function in nickel transport (19) (Fig. 4A). Thus, we focused our attention on *sven_5150–53* and *sven_4759*–*63*, the two highly expressed clusters, to assess their contributions to exploration and low-iron adaptation.

**FIG 5:**
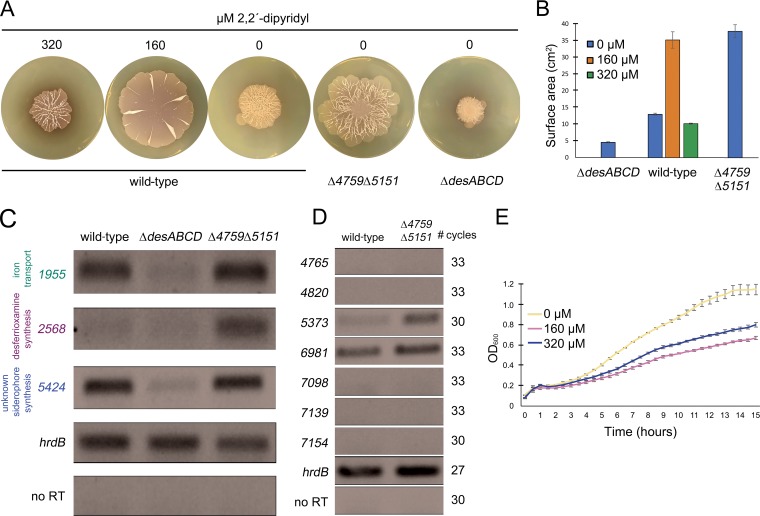
Iron uptake capabilities impact exploration. (A) Wild-type S. venezuelae exploring on YP media with 0, 160, or 320 µM dipyridyl, alongside the *desABCD* mutant and the Δ*sven_4759* Δ*sven_5151* double mutant growing on YP agar medium. Plates were incubated for 10 days. Images are representative of three replicates per strain and dipyridyl concentration (where appropriate). (B) Quantification of colony expansion by wild-type S. venezuelae, the *desABCD* mutant, and the Δ*sven_4759* Δ*sven_5151* double mutant on YP agar, with 0 to 320 µM dipyridyl for the wild type. Values represent means ± standard errors of results from three replicates. (C and D) Semiquantitative RT-PCR using RNA isolated from wild-type, Δ*desABCD* (in panel C), and Δ*sven_4759* Δ*sven_5151* double mutant strains grown for 8 days on YP medium. Vegetative sigma factor *hrdB* served as a positive control for RNA loading and RNA integrity, and no-RT reactions (performed using RNA as the template with *hrdB*-specific primers) were included as negative controls to ensure a lack of DNA contamination of RNA samples and all PCR reagents. The number of PCR amplification cycles was optimized to ensure that the products were in the linear amplification range and that no products were observed in the negative control for each reaction. For panel C, where the expression of other siderophore-associated/iron transport genes were assessed, 30 cycles were conducted for all reactions, apart from *hrdB*, where 27 cycles were used. In panel D, where expression of *bldK*-like clusters were assessed, cycle numbers for each gene are shown to the left. For both C and D, representative results are shown for experiments conducted in biological duplicate and technical triplicate. (E) Growth curves of wild-type S. venezuelae grown in liquid YP medium with 0 to 320 µM dipyridyl over 15 h. Values represent means ± standard errors of results from three replicates.

To test whether the activity of these transporters affected exploration, we constructed mutations in key genes within each of the two upregulated *bldK-*like gene clusters (*sven_4759* and *sven_5151*). To account for the possibility of functional redundancy shared by these two transporters, we also created a double (*sven_4759*, *sven_5151*) mutant strain. We found that the single mutant strains each behaved like the wild-type strain on YP (exploration-promoting) medium (Fig. S4). However, the surface area of the double mutant was significantly (approximately three times) larger than that of the wild-type strain (Fig. 5A and B). This suggested that *sven_4759* and *sven_5151* were functionally redundant and that their collective activity profoundly influenced exploration. The enhanced exploration capabilities observed for the double mutant could be reduced to wild-type levels by complementation with a cosmid carrying an intact *sven_4759–63* operon (Fig. S5).

10.1128/mBio.00171-19.4 FIG S4 Mutations within individual *bldK-*like gene clusters do not impact exploration. The wild-type S. venezuelae, Δ*sven_4759*, and Δ*sven_5151* strains were grown on YP medium. Plates were incubated for 10 days. Images are representative of three replicates per strain per condition. Download FIG S4, TIF file, 4.9 MB. Copyright © 2019 Jones et al. 2019 Jones et al. This content is distributed under the terms of the Creative Commons Attribution 4.0 International license.

10.1128/mBio.00171-19.5 FIG S5 Complementation of the double *bldK-*like mutant. Wild-type S. venezuelae, the double *bldK* mutant, and the double *bldK-*like mutant complemented with a cosmid carrying the wild-type sequence of *sven_4759* were grown on YP medium for 10 days. Download FIG S5, TIF file, 5.4 MB. Copyright © 2019 Jones et al. 2019 Jones et al. This content is distributed under the terms of the Creative Commons Attribution 4.0 International license.

These transporters were predicted to function in the uptake of iron-complexed siderophores, and their loss enhanced exploration. This led us to test whether low iron levels could generally enhance exploration. We grew wild-type S. venezuelae on YP medium supplemented with increasing concentrations of the dipyridyl iron chelator. The surface area of exploring S. venezuelae on medium containing 160 µM dipyridyl was 2.7 times larger on average than that of colonies grown without chelator (Fig. 5A and B). This was consistent with the effect that we observed for the transporter mutants (on YP without dipyridyl), supporting our proposal that these transporters were involved in iron acquisition. Doubling the chelator concentration, however, resulted in wild-type explorer colony sizes that were slightly smaller than those seen in the absence of chelator, suggesting that there was a threshold level of iron required to promote robust exploration.

These observations prompted us to create a desferrioxamine mutant strain (mutant Δ*desABCD*). We expected to see increased exploration for this mutant, as we had for the transporter mutant. Instead, we observed reduced exploration for the *des* mutant relative to the wild-type strain (Fig. 5A and B), suggesting that the level of iron uptake by this strain may fall below the threshold needed for efficient exploration.

Given the conflicting phenotypic observations for the transporter and *desABCD* mutant strains, we wondered whether these strains might differentially express other siderophores or transporters (although the *desABCD* genes were not upregulated during exploration in the wild type, expression of the genes flanking this operon was significantly enhanced, and we assume that altered expression patterns in different exploring strains would reflect biologically meaningful changes). S. venezuelae harbors five biosynthetic gene clusters with sequence similarity to clusters encoding molecules with siderophore capabilities (as predicted by antiSMASH [20]; see Table S3 in the supplemental material). While it is unlikely that all of the associated molecules function as siderophores, we assessed transcript levels for genes associated with each cluster. We isolated RNA from the wild type and from the transporter and *desABCD* mutant strains after 8 days of exploration. We analyzed the expression profiles of the genes predicted to direct siderophore production and of those predicted to be involved in siderophore uptake in these strains using semiquantitative reverse transcription-PCR (RT-PCR). Notably, S. venezuelae lacks an obvious desferrioxamine transporter: the *desE* transporter-encoding gene found adjacent to the *desABCD* cluster in S. coelicolor is missing from this gene cluster in S. venezuelae. Thus, we instead assessed the expression of *sven_3150*, which encodes a protein with the greatest sequence similarity to DesE from S. coelicolor (70% sequence similarity). Its transcript levels were essentially unchanged in both the wild-type and mutant (*desABCD* and double-transporter) strains (Fig. S6). Transcript levels of genes adjacent to the desferrioxamine biosynthetic cluster (*sven_2568*) were higher in the double-transporter mutant than in the wild type (Fig. 5C). In contrast, genes in the four other putative siderophore-encoding biosynthetic clusters were unaltered in the wild-type and transporter mutant strains (Fig. S6). Interestingly, the *desABCD* mutant exhibited reduced expression of several other transporter genes and predicted siderophore biosynthetic clusters compared with the levels seen with the wild-type and the transporter mutant strains (Fig. 5C). This altered expression may serve to further magnify the iron uptake defects of this strain and explain its reduced capacity to explore.

10.1128/mBio.00171-19.6 FIG S6 Expression profiles of genes involved in siderophore synthesis and iron uptake using semiquantitative RT-PCR. (A) RNA was isolated from wild-type, *desABCD* mutant, and Δ*sven_4759* Δ*sven_5151* double mutant strains grown for 8 days on YP medium, with “a” and “b” representing independent RNA samples for each strain. (B) RNA was isolated from wild-type and Δ*sven_4759* Δ*sven_5151* double mutant strains, as described above. For panels A and B, the SVEN numbers for the genes tested are shown on the left, alongside their associated biosynthetic cluster/predicted function (B). To the right of each panel are shown the numbers of PCR cycles used in assessing the transcript levels for each gene (ensuring that each was in the linear phase of amplification). The vegetative sigma factor *hrdB* gene served as a positive control for RNA loading and RNA integrity, and no-RT reactions (using RNA as the template with *hrdB*-specific primers) were included as negative controls to ensure a lack of DNA contamination of RNA samples and of all PCR reagents. Representative results are shown for experiments conducted in biological duplicate and technical triplicate. Download FIG S6, TIF file, 6.4 MB. Copyright © 2019 Jones et al. 2019 Jones et al. This content is distributed under the terms of the Creative Commons Attribution 4.0 International license.

We also examined the expression levels of the other *bldK*-like genes in the double-transporter mutant strain to determine whether any of these might be upregulated such that their products could compensate for the loss of the two transporters. We found that transcript levels for genes in most clusters were undetectable, with the exception of the *sven_6981-*containing cluster, whose expression was unchanged between the wild-type strain and the mutant strain, and the *sven_5373*-containing cluster, whose expression was upregulated in the double mutant background (Fig. 5D). We speculate that the transporter encoded by the latter cluster may facilitate low-level ferrioxamine uptake in the absence of the two primary transporters, ensuring sufficient intracellular iron levels for robust exploration to occur.

Finally, we sought to understand more about the mechanism underlying the increased explorer colony size observed for the siderophore transporter mutant and the wild-type strain growing under low-iron conditions. We reasoned that the surface area observed for these strains, which was larger than that observed for the wild-type strain grown without chelator, could have been due to enhanced exploration or could have been a result of increased growth. To differentiate between these possibilities, we grew wild-type S. venezuelae for 15 h in YP liquid medium, with or without the dipyridyl chelator. We found that chelator supplementation led to generally reduced S. venezuelae growth (Fig. 5E). This suggested that the enhanced exploration observed for the wild-type strain growing on 160 µM dipyridyl, as well as for the transporter mutant strain, was most likely the result of increased colony expansion and not of more-rapid growth.

### Low-iron environments can be created by interspecies interactions.

*Streptomyces* live alongside many other bacteria and fungi in soil, where there is intense competition for key nutrients, including iron. Given that low iron availability both reduced the survival of other microbes and enhanced *Streptomyces* exploration, we wondered how microbial competition for iron would impact S. venezuelae exploration. We focused our attention on interactions between *Streptomyces* and *Amycolatopsis* bacteria. These bacteria have been isolated from the same soil samples, suggesting that they may well interact in the environment (21). Furthermore, previous work has demonstrated that *Amycolatopsis* sp. AA4 can both produce its own siderophores (22) and pirate desferrioxamine E from S. coelicolor in low-iron environments (21).

**FIG 6:**
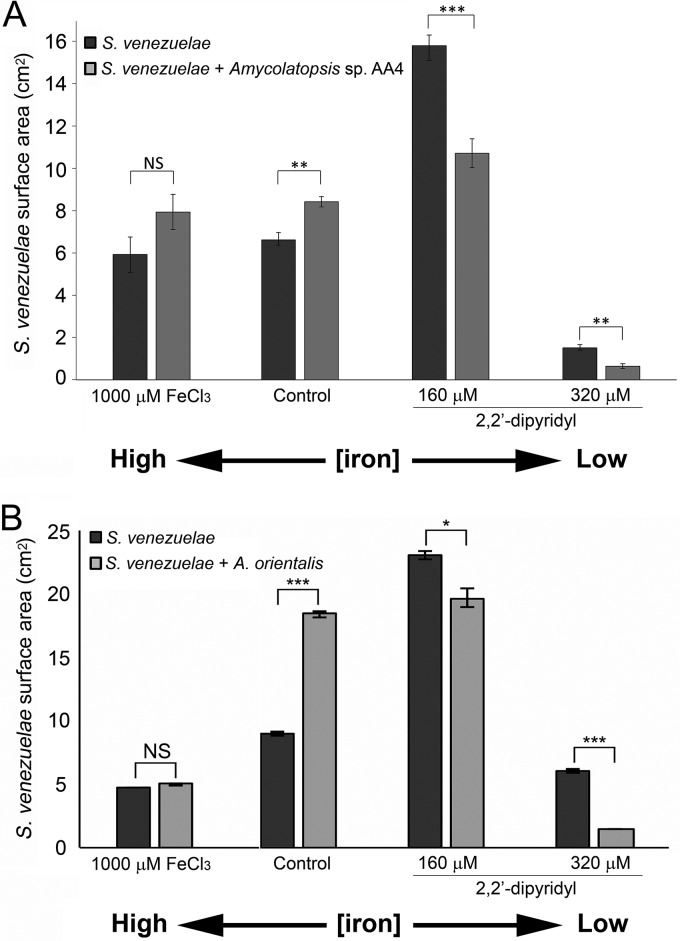
New interspecies interactions alter exploration by creating low-iron environments. (A and B) Quantification of S. venezuelae grown alone or beside (A) *Amycolatopsis* sp. AA4 or (B) A. orientalis for 7 days on YP agar supplemented with FeCl_3_ (left) or dipyridyl (right). Control: S. venezuelae grown on YP medium alone (no added iron or chelator). All values represent means ± standard errors of results from three or four replicates. Asterisks indicate statistically significant differences (*, *P* values of 0.05 to 0.01; **, *P* values of 0.01 to 0.005; ***, *P* values below 0.005; NS, no significant difference) as determined by a Student's *t* test.

We tested whether iron sequestration by two different *Amycolatopsis* spp. could affect S. venezuelae exploration. We grew S. venezuelae beside *Amycolatopsis* sp. AA4 or Amycolatopsis orientalis on either YP agar or YP supplemented with dipyridyl. Following a 7-day incubation, we found that the surface area of S. venezuelae grown beside either *Amycolatopsis* species on YP agar without chelator had increased significantly (∼30% to 100%) (Fig. 6). This was analogous to the growth of S. venezuelae alone on YP medium supplemented with moderate (160 µM) concentrations of chelator (Fig. 6). Interestingly, combining the presence of proximal *Amycolatopsis* species with further iron sequestration (through the addition of 160 or 320 µM dipyridyl) led to a decrease in the surface area of exploring S. venezuelae, suggesting that the iron levels present under these conditions were below the threshold required for optimal exploration.

Our results suggested that the presence of both *Amycolatopsis* species promoted increased S. venezuelae exploration in the same way as low-iron growth conditions. To test this hypothesis, we inoculated S. venezuelae beside the same two *Amycolatopsis* species on YP medium supplemented with up to 1,000 μM FeCl_3_ for 7 days (Fig. 6). We found that the surface areas of S. venezuelae were nearly identical under conditions of growth alone and growth beside *Amycolatopsis* species, in the presence of additional iron. This indicated that the exploration-promoting effects of *Amycolatopsis* could be suppressed by excess iron and suggested that iron sequestration by these *Amycolatopsis* species may be responsible for enhancing S. venezuelae exploration.

### Glucose trumps iron in the hierarchy of nutrients affecting exploration.

Our results to this point suggested that low iron levels, irrespective of whether such levels were due to VOC-mediated alkalinization, iron uptake defects, or iron sequestration by other organisms, resulted in enhanced exploration. This led us to question whether low iron could overcome the exploration-inhibitory effects observed for other nutrients. Previous work revealed that exploration was glucose repressible (11), and thus, we wanted to determine whether low-iron conditions could alleviate the need for a low-glucose environment. We grew S. venezuelae and the Δ*sven_4759* Δ*sven_5151* double mutant strain on plates with YP plus glucose (YPD) supplemented with a 160 µM or 320 µM concentration of the dipyridyl iron chelator for 10 days. We found that S. venezuelae failed to explore under these conditions (Fig. S7). This indicated that the presence of glucose was sufficient to override the effects of iron deficiency with respect to exploration promotion.

10.1128/mBio.00171-19.7 FIG S7 Glucose represses exploration, even in the presence of low levels of iron. Wild-type S. venezuelae and the Δ*sven_4759* Δ*sven_5151* mutant strain were grown on 0 or 160 µM 2,2'-dipyridyl. Two replicates of each strain were spotted on each plate, and the strains were incubated for 10 days. Download FIG S7, TIF file, 2.7 MB. Copyright © 2019 Jones et al. 2019 Jones et al. This content is distributed under the terms of the Creative Commons Attribution 4.0 International license.

**FIG 7:**
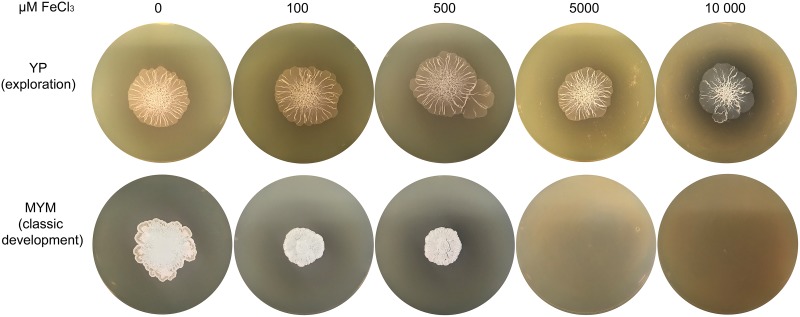
S. venezuelae growth on medium supplemented with iron. Wild-type S. venezuelae grown on YP medium (top row; exploration-promoting) or MYM medium (bottom row; classic growth medium) supplemented with 0 to 10,000 µM FeCl_3_. Images are representative of three replicates per medium type per iron concentration.

We also tested whether the addition of iron could inhibit exploration. We grew wild-type S. venezuelae on YP medium (exploration-promoting medium) and MYM medium (classic development-promoting medium) with increasing concentrations of iron (Fig. 7). S. venezuelae explored on YP medium with iron levels ranging from 0 to 10 mM, although its surface area was reduced slightly at the highest iron levels compared to the area seen with strains grown without added iron. Notably, S. venezuelae failed to grow on MYM medium when iron levels exceeded 2.5 mM, but exploring cultures remained viable during growth on concentrations of at least four times that amount. This raised the interesting possibility that exploration might be able to protect S. venezuelae from otherwise toxic levels of iron in their environment by reducing the amount of bioavailable iron through environmental alkalinization.

## DISCUSSION

Our work here reveals a new role for bacterial VOCs in modulating nutrient availability and microbial community behavior and expands knowledge of the repertoire of interspecies interactions that can impact exploration. We show that the release of TMA by *Streptomyces* explorer cells dramatically altered the pH of their surrounding environment and, in doing so, reduced the survival of nearby soil microbes by starving them of iron. Exploration is enhanced within these iron-depleted niches, and it appears that *Streptomyces* bacteria ensure maximal iron uptake by secreting siderophores and by upregulating genes encoding putative desferrioxamine transport systems. These results suggest that exploration may be not only an effective mechanism for dealing with competition for iron and iron toxicity but also has the potential to serve as a potent mediator of nutrient availability in the environment.

### Volatile compounds impact microbial community dynamics by controlling nutrient availability.

*Streptomyces* exploration is coordinated by the volatile molecule TMA, whose release raises the pH of the surrounding environment (11). Remarkably, TMA functions as both a communication cue, inducing other *Streptomyces* to explore, and a competitive weapon, reducing the survival of other microbes. The antibacterial and antifungal properties of TMA appear to be tied to its nutrient modulatory effects. Iron availability is affected by environmental pH, and alkaline conditions can create an inhospitable environment for many microbes by reducing the levels of bioavailable iron (13, 14, 23). We show that iron supplementation can restore the growth of otherwise susceptible bacteria and fungi in the presence of TMA, suggesting that iron starvation is at the heart of the TMA antimicrobial effects.

Microbes can modify their environment through nutrient depletion or through metabolite excretion and, in doing so, can impact the growth and dynamics of their surrounding community members. Volatile compounds are being increasingly recognized as important contributors to soil nutrient status. For example, microbial volatiles can generally impact carbon dynamics in the soil (24). Species-specific volatiles have also been shown to enhance the availability of reduced sulfur, which in turn promotes the growth of sulfur-deficient plants (25). We can now add volatile-induced iron starvation through increased environmental pH to the effects ascribed to microbial volatiles.

### Promoting exploration in iron-depleted environments.

**FIG 8:**
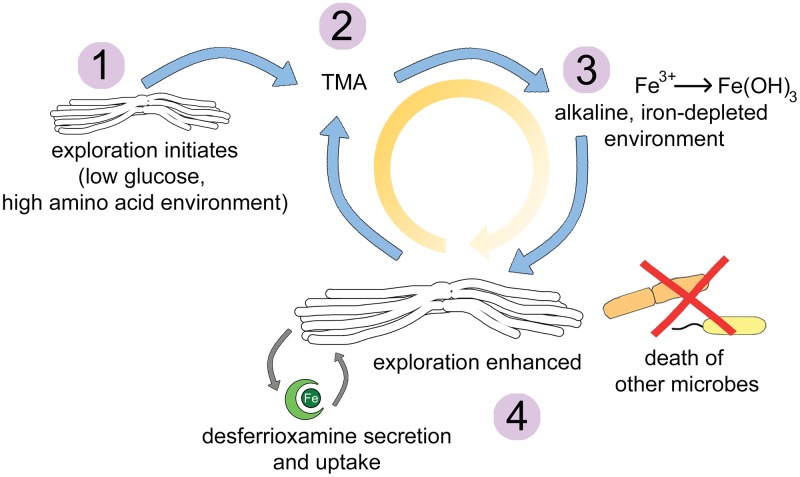
Working model for how S. venezuelae explorer cells thrive and impact the survival of other microbes in alkaline, low-iron environments. Step 1: S. venezuelae exploration is triggered by a combination of low glucose and high amino acid concentrations. Step 2: explorer cells release the VOC TMA into the surrounding environment. Step 3: TMA raises the pH of the environment, concomitantly reducing the solubility and bioavailability of iron. Step 4: to cope with low-iron conditions, S. venezuelae explorer cells release desferrioxamines. These siderophores return solubilized iron to the cells. At the same time, S. venezuelae exploration is enhanced, perhaps as a mechanism to reach environments richer in iron. Enhancing exploration leads to increased TMA production, and this may create a positive-feedback loop (circular arrow) where TMA depletes iron, explorer cells spread to get more iron, TMA production continues, and the cycle repeats. Within these alkaline, iron-depleted environments, the growth of other bacteria and fungi is reduced.

*Streptomyces* exploration is enhanced during growth in low-iron environments, although below a certain level, exploration stimulation ceases. Iron is limited in the soil (21, 26), and exploratory growth in iron-limited environments could enable *Streptomyces* to access nutrients in more-distant locations. We have determined that exploration is both rapid and remarkably processive; explorer cells spread over surfaces at a rate that is ∼12 times faster than has been seen previously for *Streptomyces* colonies (11), and it is not yet known how exploration is stopped. Our data suggest that the relentless nature of exploration may be driven by a positive-feedback loop, with iron acting as a central player. Our working model involves explorer cells producing TMA, with TMA emissions leading to increased pH and reduced iron availability. Explorer cells then spread to get more iron, leading to the continued production of TMA, and thus continued exploration (Fig. 8). Support for this model has come from a recent study modeling the effects of pH on bacterial growth. That work demonstrated that when an environmental modification is beneficial for a bacterium, there is positive feedback for their growth; the more they change the environmental pH, the more cells grow, and the more they continue to alter pH (23).

Within this putative exploration feedback cycle, several additional factors could account for the increase in exploration surface area in low-iron environments. The classic *Streptomyces* life cycle involves a transition from vegetative hyphal growth to raising reproductive aerial structures. For S. coelicolor, adding iron chelators to the growth medium prevents colonies from raising aerial hyphae or sporulating and instead locks cells in the vegetative-growth phase (21). Explorer cells share many properties with vegetative hyphae (11), and it is possible that reduced iron availability helps to inhibit aerial development, thereby enhancing the rate of exploration by preventing entry into the classical reproductive differentiation phase. It has also been reported that desferrioxamines, like those produced by exploring cultures, can function as morphogens (18, 27); it will be interesting to see whether these molecules contribute to exploration beyond their roles in iron acquisition.

Iron levels, in addition to modulating the rate of *Streptomyces* exploration, also influenced the architecture of the exploring colonies. Colonies growing on YP supplemented with the dipyridyl chelator were much flatter and less structured than colonies growing alone on YP. Other bacteria (e.g., Pseudomonas aeruginosa) require iron for biofilm assembly, where low iron triggers increased surfactant production and motility, which in turn reduce biofilm structure (28, 29). Similar connections between iron availability and motility have been made in other microbes (30–33). *Streptomyces* exploration is phenotypically similar to sliding motility—a form of passive motility driven by growth and facilitated by the release of a surfactant. It is possible that low iron availability enhances surfactant production by explorer cells. This could in turn alter colony architecture and promote greater motility and at the same time increase the colony’s ability to scavenge for iron.

How *Streptomyces* bacteria sense low iron levels during exploration remains unexplained. It does not appear to be mediated by the major iron repressor DesR/DmdR, as expression of the *des* cluster, a known regulon member in other streptomycetes (17, 34–36), is unaltered during exploration. It is also not clear what controls the expression of the two transporter-encoding clusters, as these do not have an iron box present in their promoter regions. It will be interesting to determine whether this response is coupled with pH sensing, given the profound effect of high pH on iron availability.

### Interspecies effects on *Streptomyces* metabolism and exploration.

*Streptomyces* exploration was initially discovered as a response to coincubation with the yeast S. cerevisiae (11). Specifically, S. cerevisiae depletes glucose from the medium, and this change in carbon source availability seems to promote the onset of exploration (11). We show here that organisms such as *Amycolatopsis* that are capable of “stealing” siderophores and otherwise sequestering iron can also influence the rate of exploration. Intriguingly, S. venezuelae secretes a suite of differentially modified siderophores during exploration. Desferrioxamine diversification is not unprecedented. In Streptomyces chartreusis, distinct desferrioxamines are produced under different growth conditions (37), and similar desferrioxamine diversification patterns have been seen in S. coelicolor grown in association with different actinomycetes (21). It will be interesting to determine whether some of these molecules are more amenable for uptake by S. venezuelae than by other organisms and whether these modified compounds provide resistance against siderophore cheaters. Alternatively, the high-pH environment may promote differential siderophore tailoring, as has been noted for marine microbes that produce more-amphiphilic ferrioxamines (38). In exploring S. venezuelae, this may be the result of increased expression of the genes flanking the core *desABCD* operon and/or of the effect of modulating the availability of different precursors.

Collectively, our results suggest that exploring *Streptomyces* colonies can alter nutrient availability through volatile compound emission. We propose that this nutrient modulation may activate a positive-feedback cycle that promotes continued exploration. This in turn, would have the potential to further drive changes in the dynamics and composition of the local microbial community.

## MATERIALS AND METHODS

### Strains, plasmids, media, and culture conditions.

Strains, plasmids, and primers used in this study are listed in Table S1 in the supplemental material. S. venezuelae ATCC 10712 was grown on MYM (maltose, yeast extract, malt extract) agar for spore stock generation and for examination of the behavior of classically developing cultures. Exploration was investigated on YP (yeast extract, peptone) agar or in association with yeast on YP agar supplemented with dextrose/glucose (YPD). Nonexploring controls were grown by themselves on YPD agar. For iron experiments, plates were supplemented with the indicated concentration of 2,2'-dipyridyl (0 to 360 µM) or FeCl_3_ (0 to 10 mM). All strains were grown at 30°C, except the strains used in the experiments involving TMA, which were conducted at room temperature in a fume hood. Prior to growing on plates, S. venezuelae was grown in liquid MYM medium at 30°C, and 5-µl volumes were spotted onto agar plates. *Amycolatopsis* sp. were also grown in liquid MYM medium at 30°C, and 5 µl of the overnight culture was spotted alone or directly beside S. venezuelae onto the surface of YP agar medium. All plates were incubated for up to 14 days. Colony surface areas were measured using ImageJ (39). Escherichia coli strains were grown in or on LB (lysogeny broth) medium or in SOB (Super Optimal Broth) medium. DH5α and ET12567/pUZ8002 strains were grown at 37°C, and BW25113/pIJ790 was grown at 30°C or 37°C.

10.1128/mBio.00171-19.8 TABLE S1 Bacterial strains and plasmids. Download Table S1, DOCX file, 0.02 MB. Copyright © 2019 Jones et al. 2019 Jones et al. This content is distributed under the terms of the Creative Commons Attribution 4.0 International license.

For iron growth assays, B. subtilis and M. luteus were grown in LB medium, while S. cerevisiae was grown in YPD medium. Each strain was grown overnight in 10 ml liquid medium at 30°C with shaking. To quantify strain survival in response to iron chelation on solid medium, each strain was subcultured and grown to an OD_600_ of 0.8, before being diluted 1/5,000, with 100 µl being spread on agar medium containing 0 to 360 μM 2,2’ dipyridyl. Colony numbers were quantified after 2 days. To quantify the survival of each strain in response to iron chelation in liquid medium, various amounts of overnight cultures of each strain were added to 0.8 ml fresh media to give an OD_600_ of 0.1 in 48-well plates. Plates were shaken at 30°C in a plate reader, and OD_600_ readings were taken every 30 min for 8 h. To ensure that the effects of 2,2′ dipyridyl were specific for iron chelation, 2 mM FeCl_3_ was also added to the subcultures, which were then grown as described above.

### Assays for volatile-mediated phenotypes.

To quantify how S. venezuelae VOCs affected the survival of other bacteria or fungi, S. venezuelae was grown in a small petri dish containing YP or YPD agar. The small dish was placed inside a larger dish containing agar or agar supplemented with 1 mM FeCl_3_. LB agar was used for the growth of B. subtilis and M. luteus, while YPD agar was used for S. cerevisiae. S. venezuelae*-*inoculated plates were grown for 10 days, after which the indicator organisms were spread on the surrounding plates. B. subtilis, M. luteus, and S. cerevisiae were subcultured and grown to an OD_600_ of 0.8 in liquid LB/YPD, after which the cultures were diluted 1/10,000 and 50-µl volumes were then spread on the agar. Colony numbers on the outer plate were quantified after 48 h. Note that the *Streptomyces* cultures and the indicator strains were physically separated, preventing the exchange of anything other than volatile compounds between organisms.

Measuring how iron supplementation affected the responses of microbes to TMA involved adding either 1.5 ml of commercially available TMA solutions (Sigma) diluted to 0.9% to 5% (wt/vol) or water (negative control) to sterile plastic containers. These were then placed in a petri dish containing 50 ml LB or YPD agar, with or without 1 mM FeCl_3_ supplementation. The effects of the presence of manganese and copper were assessed in an identical way, using MnCl_2_ or CuCl_2_ supplemented at 1 mM. B. subtilis, M. luteus, and S. cerevisiae were subcultured and grown to an OD_600_ of 0.8 in liquid LB/YPD before 100 μl of each subculture was spread on each plate. Plates were incubated at room temperature for 48 h, after which cells were scraped into tubes containing 2 ml liquid LB or YPD and vigorously mixed. Dilution series were used to measure the OD_600_ of the resulting cell suspensions. A minimum of four biological replicates were assessed, alongside two technical replicates in each instance.

### Identification and analysis of desferrioxamines.

To analyze metabolite production by explorer cultures, the contents of each plate (agar plus cells), alongside those of a medium-alone control, were diced, placed in a 50-ml Falcon tube, and frozen at −80°C. The culture or agar was then dried by lyophilization. Extraction of metabolites was performed by addition of 45 ml of n-butanol/ethyl acetate (50:50) to each Falcon tube and rotation overnight at room temperature. The extracts were filtered, and the solvent was removed under vacuum at room temperature (Genevac EZ-2 series personal evaporator) (medium-low plus medium boiling points [BP], lamp off).

All mass spectrometry experiments were conducted using ultrapressure liquid chromatography-electrospray ionization-quad time of flight mass spectrometry (UPLC-ESI-QTOF MS) instrumentation (Agilent catalog no. 6540), as described previously (40, 41). Each extract was dissolved in 400 µl of H_2_O/acetonitrile (Fisher; LC-MS grade) (50:50), 2 µl of which were separated on a Zorbax C_18_ column (Agilent) (2.1 by 50 mm, 1.8 µm pore size). Compounds were separated using a constant flow of 0.4 ml/min and the following gradient: 0 to 3 min at 0% buffer B (buffer A, 95%:5%:0.1% H_2_O/acetonitrile [ACN]/formic acid; buffer B, 95%:5%:0.1% ACN/H_2_O/formic acid), 3 to 17 min at 0% to 100% B, and 17 to 20 min at 100% B. Accurate mass data were acquired in triplicate in both profile mode and centroid mode with a source/fragmentor voltage of 100 V, positive mode ion detection at between 100 and 1,700 *m*/*z*, gas temperature of 325°C, and capillary voltage of 3,500 V. Fragmentation data were acquired with a fixed collision energy of 35 V with positive-mode product ion detection between 50 and 1,650 *m*/*z*. Data were processed using Agilent Masshunter qualitative analysis and origin.

### Construction of deletion strains and mutant complementation.

In-frame deletions of *sven_4759* (*bldK* homolog) and *sven_2570–73* (*desA-D*) were generated using ReDirect technology (Gust et al. [42]). For each of *sven_4759* and *desA-D*, the coding sequence (from start to stop codon) was replaced with an *oriT*-containing apramycin resistance cassette. For mutation of *sven_5151* (a second *bldK* homolog), the gene was disrupted in the chromosome. A 1,406-bp region of the gene was amplified and cloned into TOPO vector (Invitrogen) in which an *oriT*-containing hygromycin resistance cassette had replaced the ampicillin resistance gene in the vector backbone. Mutant cosmids/disruption plasmids were introduced into nonmethylating E. coli strain ET12567/pUZ8002 prior to conjugation into wild-type S. venezuelae. For creating the Δ*sven_4759* Δ*sven_5151* double mutant strain, the ET12567/pUZ8002 strain carrying the *sven_5151* TOPO construct was introduced into the Δ*sven_4759*:apramycin strain. The resulting exconjugants were screened for double-crossover recombinants (for strains *sven_4759 bldK* and *sven_2570–73*) or single-crossover integration (for strains *sven_5151* and Δ*sven_4759* Δ*sven_5151*). Correct replacement of *sven_4759* or *sven_2570–73* or disruption of the *sven_5151* coding sequence was confirmed using diagnostic PCR combinations (see Table S2 for oligonucleotide sequences and use). The Δ*sven_4759* Δ*sven_5151* double mutant phenotype was complemented using a cosmid carrying the wild-type *sven_4759* sequence, along with the downstream cluster to account for any polar effects. To enable effective selection for cosmid integration into the S. venezuelae chromosome, the ampicillin resistance gene on the cosmid backbone was replaced with an *oriT*-containing viomycin resistance cassette, using the ReDirect protocol (42).

10.1128/mBio.00171-19.9 TABLE S2 Oligonucleotides used in this study. Download Table S2, DOCX file, 0.02 MB. Copyright © 2019 Jones et al. 2019 Jones et al. This content is distributed under the terms of the Creative Commons Attribution 4.0 International license.

10.1128/mBio.00171-19.10 TABLE S3 S. venezuelae siderophore production and uptake genes. Download Table S3, DOCX file, 0.01 MB. Copyright © 2019 Jones et al. 2019 Jones et al. This content is distributed under the terms of the Creative Commons Attribution 4.0 International license.

### RNA isolation and RT-PCR analysis.

RNA was isolated as described previously (43) from two replicates each of wild-type and Δ*sven_4759 Δsven_5151* S. venezuelae exploring colonies grown for 8 days on YP agar plates. For all replicates, contaminating DNA was removed using Turbo DNase (Life Technologies) and was confirmed to be DNA free by PCR. RNA quality and purity were assessed using a NanoDrop spectrophotometer. RNA integrity was further analyzed by agarose gel electrophoresis prior to reverse transcription-PCR (RT-PCR) analysis.

One microgram of RNA was used as the template for reverse transcription using gene-specific primers (see Table S2) and SuperScript III polymerase (Invitrogen), according to the manufacturer’s instructions. The resulting cDNA then served as the template for PCR amplification performed using *Taq* DNA polymerase and the gene-specific primers listed in Table S2. The number of cycles was optimized to ensure that the products were detected in the linear range of amplification. Negative controls containing nuclease-free water instead of reverse transcriptase were included to ensure that the RNA samples and other reagents did not contain residual contaminating DNA (“no-RT” controls). cDNA corresponding to the *hrdB* vegetative sigma factor was amplified as a positive control for RNA levels and RNA integrity. A 10-μl volume of each PCR was separated on a 1.5% agarose gel and visualized by staining with ethidium bromide. All reactions were conducted in triplicate, using two independently isolated RNA samples.
