# Supplementary figures and images for: Streptomyces Volatile Compounds Influence Exploration and Microbial Community Dynamics by Altering Iron Availability
Source: mBio. 2019 Mar 5;10(2):e00171-19. doi: 10.1128/mBio.00171-19 (PMC6401478; doi:10.1128/mBio.00171-19)

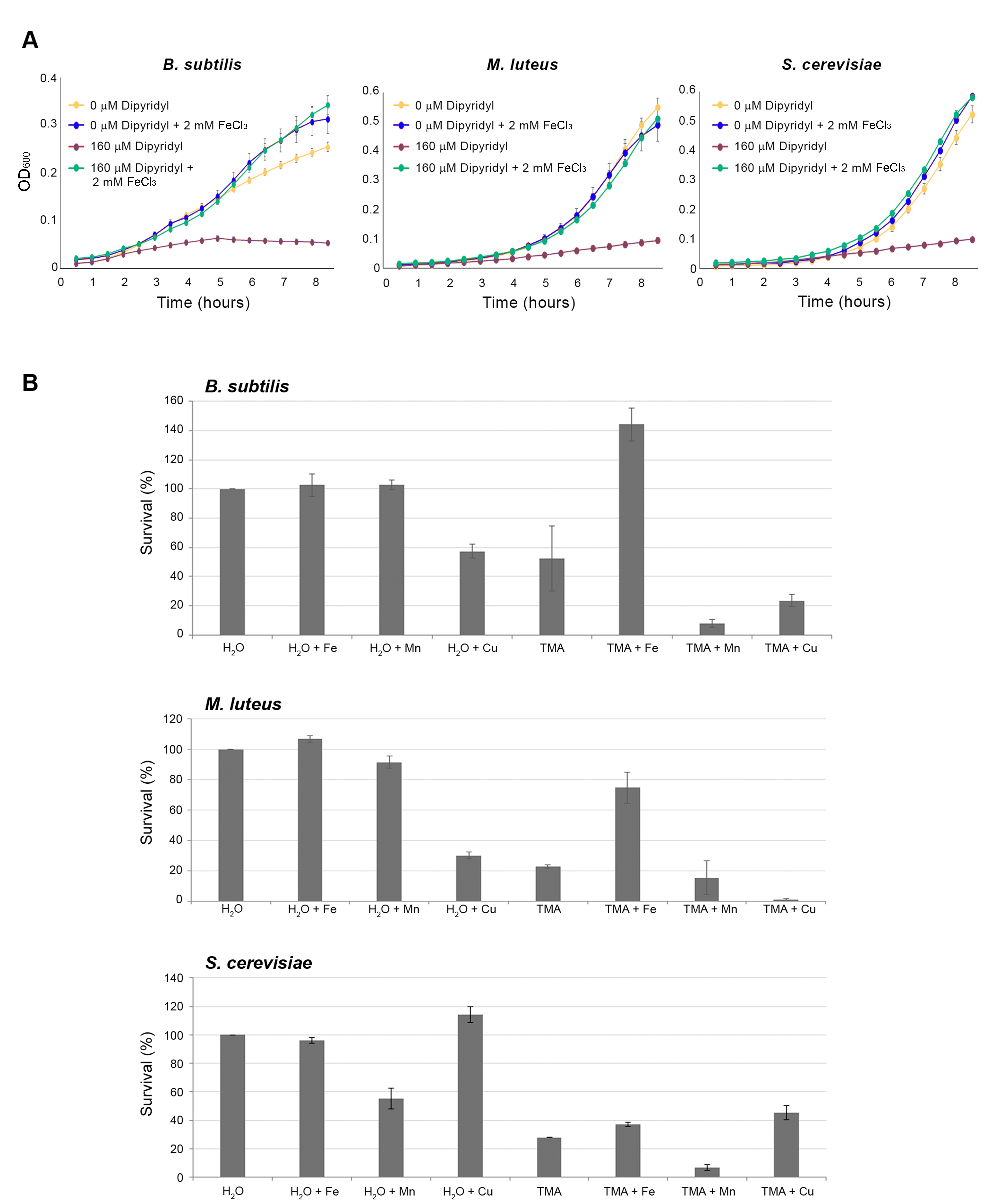

Supplement: FIG S1 [file mBio.00171-19-sf001.tif]

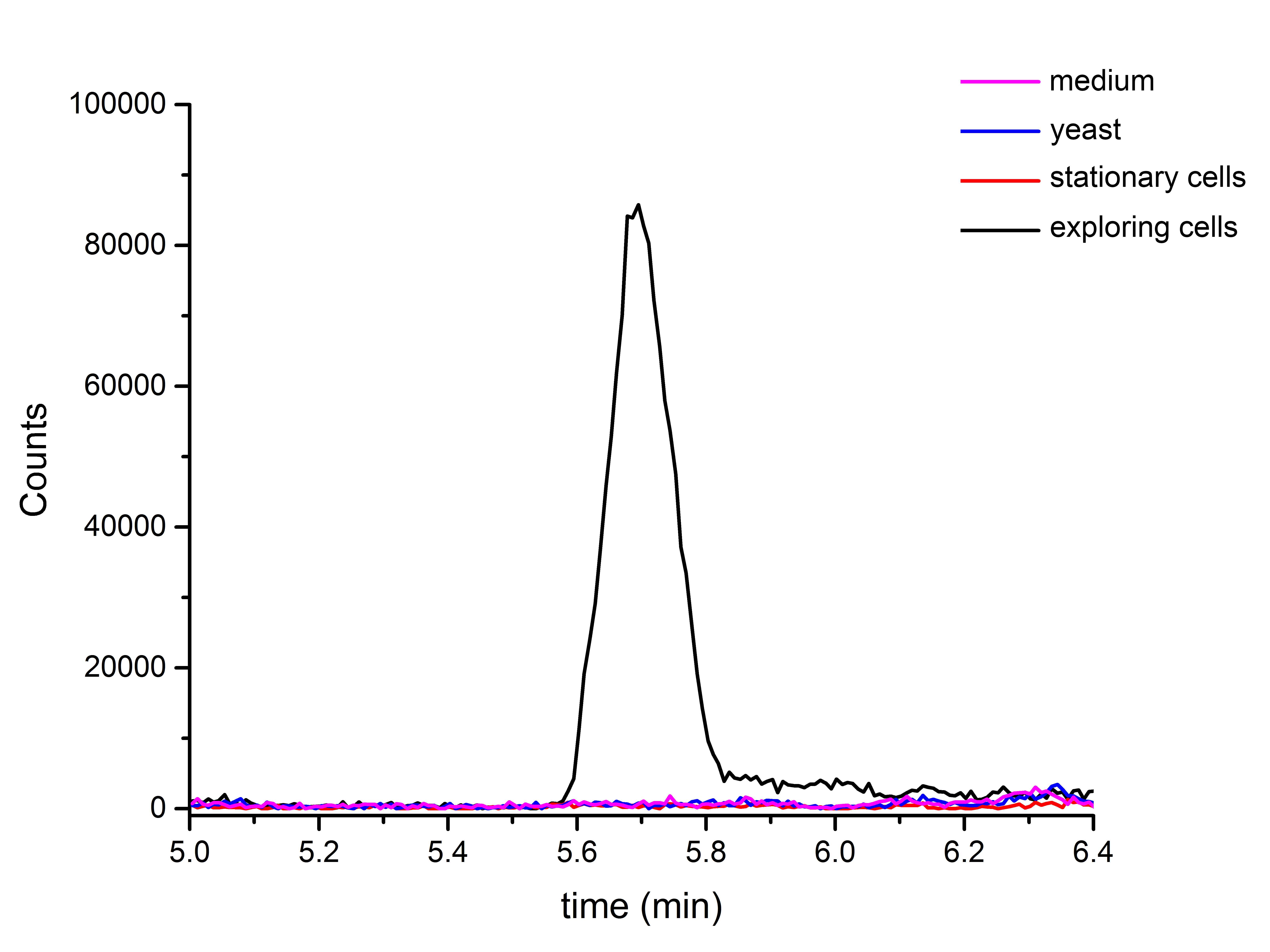

Supplement: FIG S2 [file mBio.00171-19-sf002.tif]

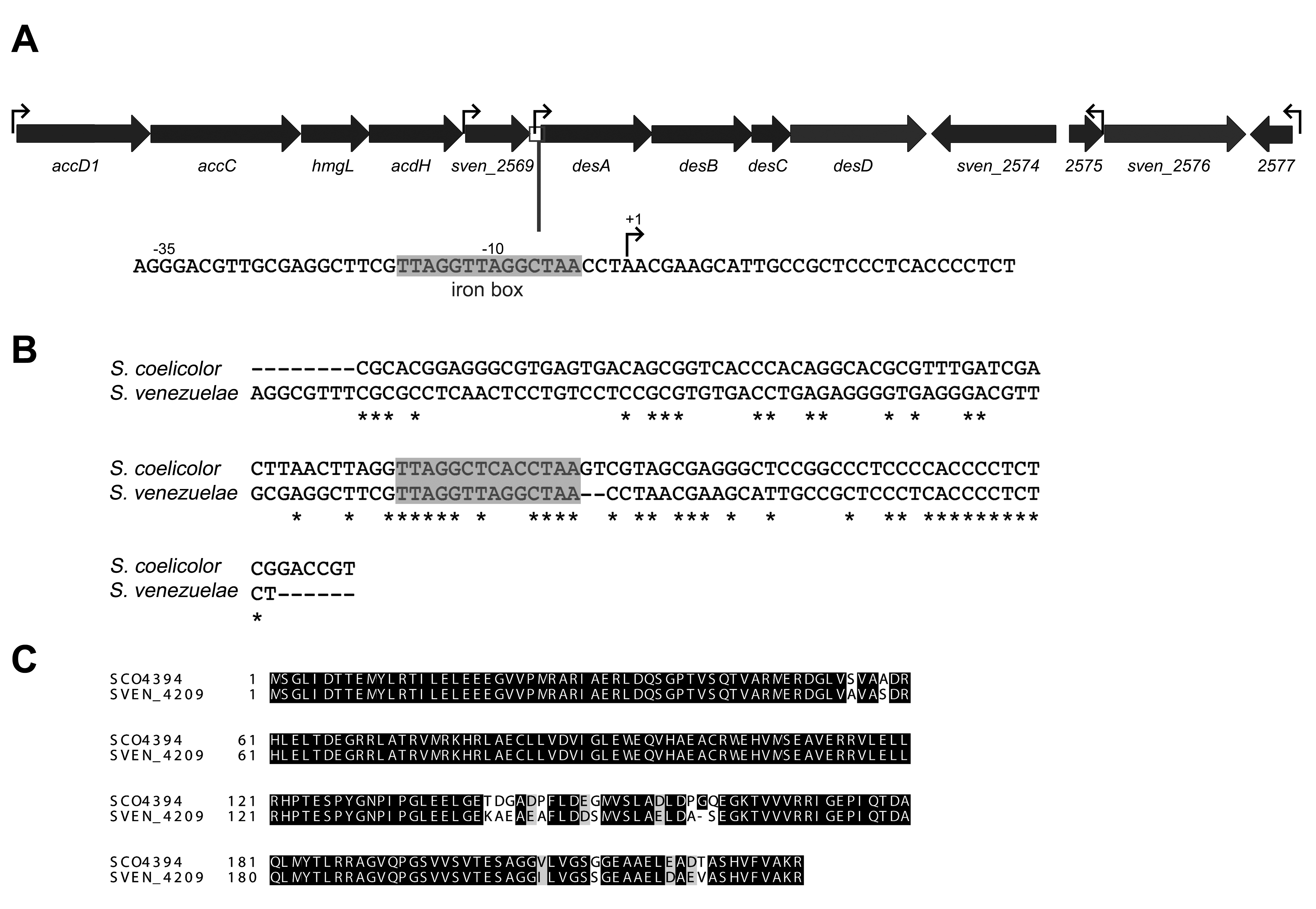

Supplement: FIG S3 [file mBio.00171-19-sf003.tif]

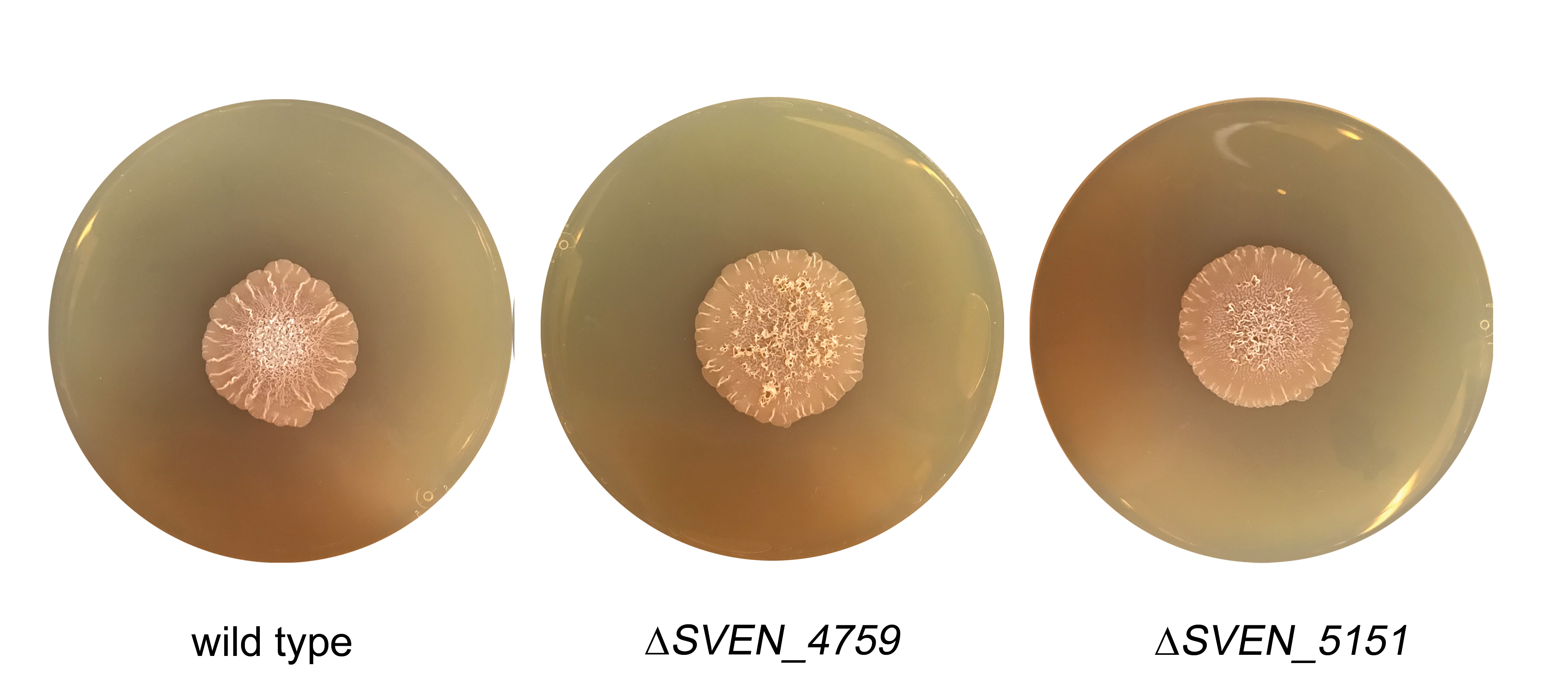

Supplement: FIG S4 [file mBio.00171-19-sf004.tif]

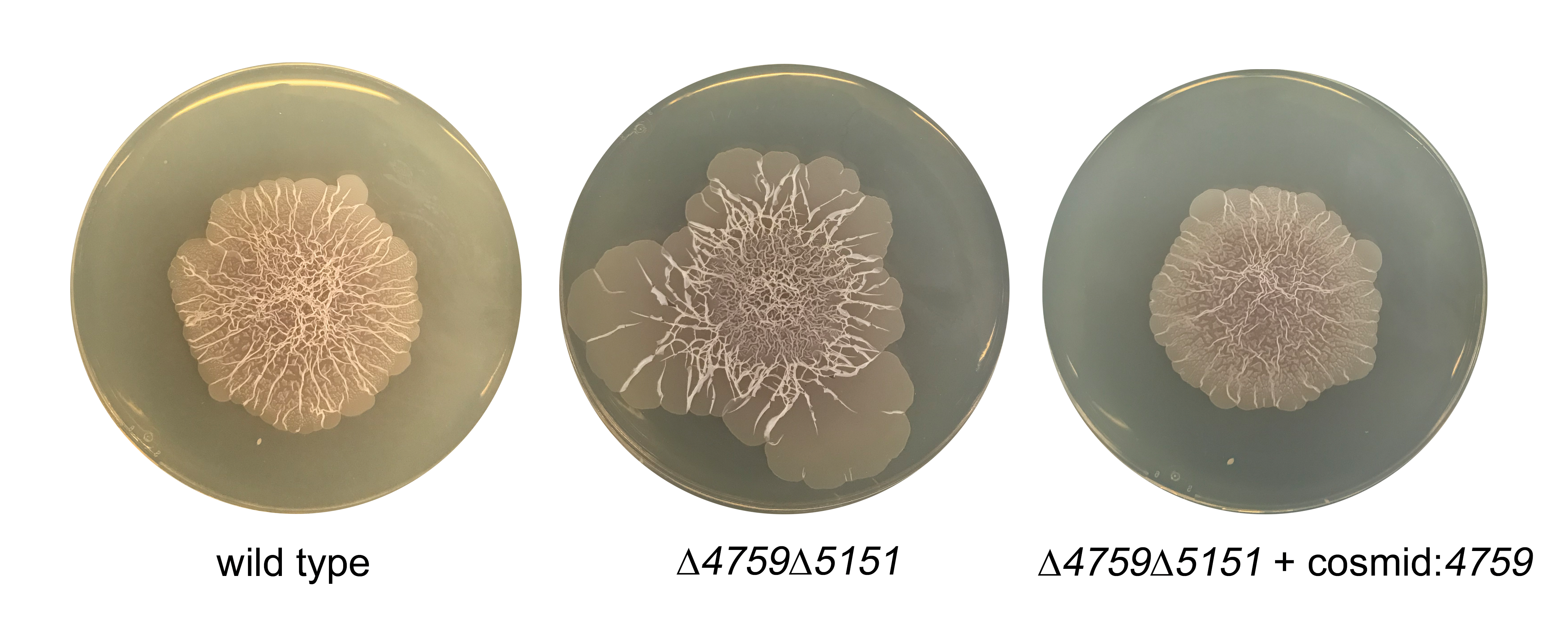

Supplement: FIG S5 [file mBio.00171-19-sf005.tif]

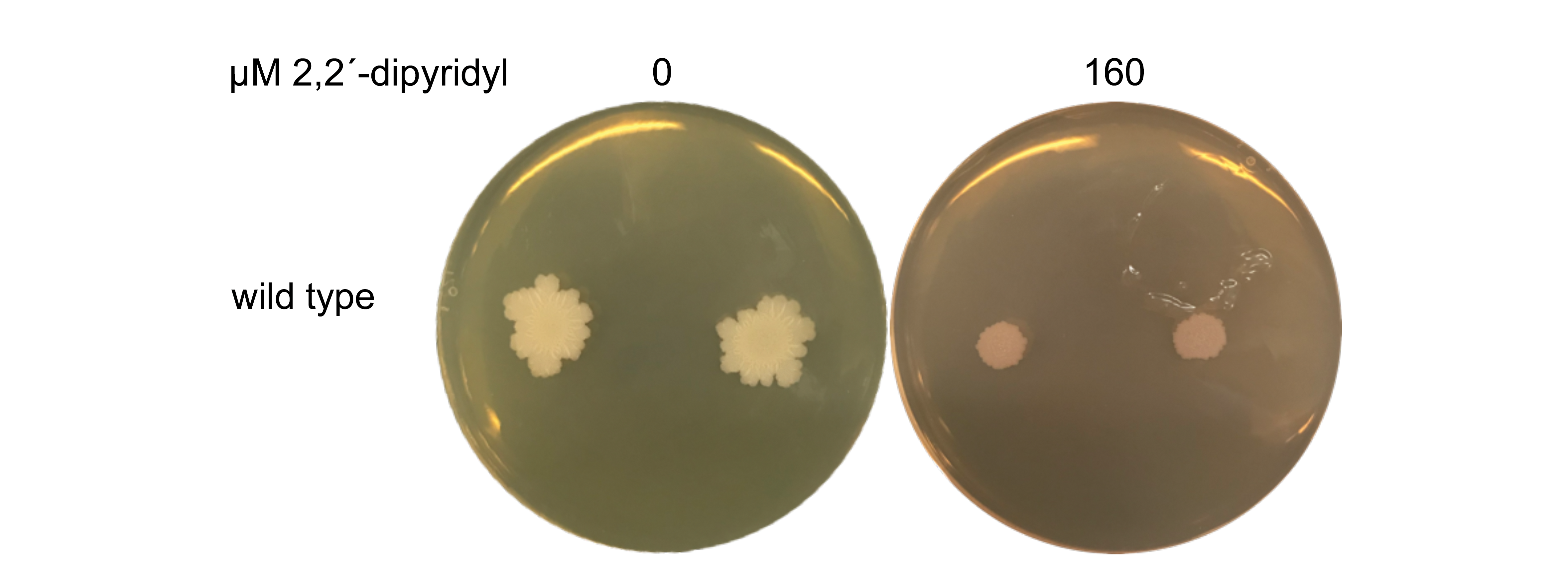

Supplement: FIG S7 [file mBio.00171-19-sf007.tif]
