# Supplementary material for: Streptomyces Volatile Compounds Influence Exploration and Microbial Community Dynamics by Altering Iron Availability
Source: mBio. 2019 Mar 5;10(2):e00171-19. doi: 10.1128/mBio.00171-19 (PMC6401478; doi:10.1128/mBio.00171-19)
Supplement: TABLE S1 [file mBio.00171-19-st001.docx]

# Table S1. Bacterial strains and plasmids

| Strain | Genotype, description, or use | Reference or source |
| --- | --- | --- |
| ***Streptomyces*** |  |  |
| *Streptomyces venezuelae*  ATTC 10712 |  | Gift from M. Buttner |
| E329 | ATTC 10712 ∆*SVEN_4759::[aac(3)IV-oriT]* | This work |
| E330 | ATTC 10712 ∆*SVEN_5151::*TOPO 2.1 | This work |
| E330 | ATTC 10712 ∆*SVEN_4759::[aac(3)IV-oriT]*  + ∆*SVEN_5151::*TOPO 2.1 | This work |
| E331 | ATTC 10712 ∆*SVEN_2570-73::[aac(3)IV-oriT]* | This work |
|  |  |  |
|  |  |  |
| ***Amycolatopsis*** |  |  |
| *Amycolatopsis orientalis*  sp. *Lurida* |  | Gift from G. Wright |
| *Amycolatopsis* sp. AA4 |  | Gift from M. Traxler |
|  |  |  |
|  |  |  |
| **Indicator strains** |  |  |
| *Bacillus subtilis* 168 |  | Gift from J. Nodwell |
| *Micrococcus luteus* |  | Gift from J. Nodwell |
| *Saccharomyces cerevisiae* BY4741 | *MAT****a****; his3*Δ*1; leu2*Δ*0 ura3*Δ*0 met15*Δ*0* | Gift from L. Cowen |
|  |  |  |
|  |  |  |
| ***E. coli*** |  |  |
| DH5α | Plasmid construction and subcloning | Invitrogen |
| ET12567/pUZ8002 | Generation of methylation-free plasmid DNA and conjugation into *Streptomyces* | (39, 40) |
| BW25113/pIJ790 | Introducing mutations in cosmid DNA | (15) |
|  |  |  |
|  |  |  |
| **Plasmids or cosmids** |  |  |
| 3D02 | *S. venezuelae* cosmid carrying *SVEN_4759* | Gift from M. Bibb and M. Buttner |
| 5-E05 | *S. venezuelae* cosmid carrying *SVEN_2570-73* | Gift from M. Bibb and M. Buttner |
| TOPO 2.1 | Plasmid used for disruption of *SVEN_5151* | Invitrogen |
| pIJ790 | Temperature-sensitive plasmid carrying λ- RED genes | (15) |
| pIJ773 | Plasmid carrying the apramycin knockout cassette | (15) |
| pIJ10701 | Plasmid carrying the *hyg-oriT* cassette | (15) |
| pIJ780 | Plasmid carrying the *vio-oriT* cassette | (15) |
