# Supplementary material for: Streptomyces Volatile Compounds Influence Exploration and Microbial Community Dynamics by Altering Iron Availability
Source: mBio. 2019 Mar 5;10(2):e00171-19. doi: 10.1128/mBio.00171-19 (PMC6401478; doi:10.1128/mBio.00171-19)
Supplement: TABLE S2 [file mBio.00171-19-st002.docx]

**Table S2. Oligonucleotides used in this study**

| Name | Sequence (5ʹ to 3ʹ) | Use |
| --- | --- | --- |
| SVEN4759  Fwd | CTTGTAGTGACAAAGTGGACTCATGAGGAG GAACCCATGATTCCGGGGATCCGTCGACC | Creating and confirming the Δ*4759* mutation |
| SVEN4759  Rev | TCAGCCCCGCATCGCAGGCGGACTACGCTTG GGTGATTATGTAGGCTGGAGCTGCTTC | Creating and confirming the Δ*4759* mutation |
| SVEN4759  Up | TCCGGTACGTGTGTTAACGG | Confirming the ∆*4759* mutation |
| SVEN4759  Down | GGGATCATCTGGAGCAGTCG | Confirming the ∆*4759* mutation |
| SVEN4759 In | CGGCGTTGATCATCTCCAGC | Confirming the ∆*4759* mutation |
| SVEN2570-73 Fwd | TCCCTCACCCCTCTCTGCTGTCCGGAGGCCCCCCACATGATTCCGGGGATCCGTCGACC | Creating and confirming the ∆*2570-73* (*des*) mutation Confirming the ∆*2570-73* mutation |
| SVEN2570-73 Rev | CGTACTCCGGCGGGGCTCGCATCGGTCTCGGGGGGGTTATGTAGGCTGGAGCTGCTTC | Creating and confirming the ∆*2570-73* (*des*) mutation |
| SVEN2570-73 Up | ATATTCTAGAGACGAGACGCAGGAAGACGC | Confirming the ∆*2570-73* (*des*) mutation |
| SVEN2570-73 Down | GTTCGACGCCCTGGACATCG | Confirming the ∆*2570-73* (*des*) mutation |
| SVEN2570-73 In | ATATTCTAGACACACCGGTGAACGGTCCTC | Confirming the ∆*2570-73* (*des*) mutation |
| SVEN5151  Up | ATATGGTACCACTTCGCCCGCTACTACACG | Creating and confirming the Δ*5151* mutation |
| SVEN  5151 Down | ATATTCTAGAGGTGAAGGGCAGGTAGACCG | Creating and confirming the ∆*5151* mutation |
| SVEN5151  CheckF | CAACGCCTCCGACAAGAAGG | Confirming the ∆*5151* mutation |
| SVEN5151  CheckR | CTGCTTGCCGACGAACATCG | Confirming the ∆*5151* mutation |
| blaF | CCCTGATAAATGCTTCAATAATATTGAAAAA GGAAGAGTA | Amplifying the *hyg-oriT* and *vio-oriT* cassettes; cosmid-based complementation of the  ∆*4759*∆*5151* mutant strain |
| blaR | AATCAATCTAAAGTATATATGAGTAAACTTG GTCTGACAG | Amplifying the *hyg-oriT* and *vio-oriT* cassette; cosmid-based complementation of the ∆*4759*∆*5151* mutant strain |
| HrdBF | CCGTTTCCATCGTTCCGAGA | Semi-quantitative RT-PCR |
| HrdBR | ATCTGCCCATCAGCCTTTCC | Semi-quantitative RT-PCR |
| 0164F | GTGTCGTTCTCCTGGCCGG | Semi-quantitative RT-PCR |
| 0164R | GTTGTCCGCGACGACGGTG | Semi-quantitative RT-PCR |
| 0512F | TGATGACCCTCGTCAATCGG | Semi-quantitative RT-PCR |
| 0512R | CAACAATGCCTCCCAGGACC | Semi-quantitative RT-PCR |
| 0517F | CTGCACGCGGAGGAGTACG | Semi-quantitative RT-PCR |
| 0517R | GAGCAGATAGGCCGCCTGC | Semi-quantitative RT-PCR |
| 0777F | GTCGTCGAGCACCTCATCG | Semi-quantitative RT-PCR |
| 0777R | CCTTGGGCTCGTTCTTCAGC | Semi-quantitative RT-PCR |
| 1955F | TGCACCGAGAAGAGCGACG | Semi-quantitative RT-PCR |
| 1955R | CGGCGAGGTTCACGTGACC | Semi-quantitative RT-PCR |
| 1997F | GAGGAGGCGACGGAGGATCC | Semi-quantitative RT-PCR |
| 1997R | GCGTCGCCGAAGTCCTTGC | Semi-quantitative RT-PCR |
| 2568F | GAGATCGTCCGCGAGATGG | Semi-quantitative RT-PCR |
| 2568R | GAGATCGTCCGCGAGATGG | Semi-quantitative RT-PCR |
| 2570F | CCGTTCACCGGTGTGACCC | Semi-quantitative RT-PCR |
| 2570R | GGAGGTAGACGCTCTCCAGC | Semi-quantitative RT-PCR |
| 3150F | CGCGACCCGGCGAAGG | Semi-quantitative RT-PCR |
| 3150R | CTTCCCGTCGTAGGTCACG | Semi-quantitative RT-PCR |
| 4765F | CATCGTGAGTTCCTCGTGGG | Semi-quantitative RT-PCR |
| 4765R | GTCGTACTGCTTGAGACCCC | Semi-quantitative RT-PCR |
| 4820F | GCTGACCGTCCTCAACACCG | Semi-quantitative RT-PCR |
| 4820R | TGAGCGTACGGAAGACGAGG | Semi-quantitative RT-PCR |
| 5373F | TGGCCTACGAGTACCTCACC | Semi-quantitative RT-PCR |
| 5373R | GTCCACGTCAGCTTGTCCG | Semi-quantitative RT-PCR |
| 5419F | ACCGAGTCCCGACGGATCC | Semi-quantitative RT-PCR |
| 5419R | CCTCCCGTCCACAGCTCGG | Semi-quantitative RT-PCR |
| 5424F | AGGTGATCTCCACCGGATCG | Semi-quantitative RT-PCR |
| 5424R | CGTCAGGGTCGTCTTACCG | Semi-quantitative RT-PCR |
| 0164F | GTGTCGTTCTCCTGGCCGG | Semi-quantitative RT-PCR |
| 0164R | GTTGTCCGCGACGACGGTG | Semi-quantitative RT-PCR |
| 5475F | CAGCCGGGTACCACCGTCC | Semi-quantitative RT-PCR |
| 5475R | GTACTGCGGGCCGGATAGG | Semi-quantitative RT-PCR |
| 5479F | CGCCATGAATGACGCAAGC | Semi-quantitative RT-PCR |
| 5479R | GGTGAGACCGGAGCTGTCG | Semi-quantitative RT-PCR |
| 6981F | AAGGATTCCGCGGTCGTCG | Semi-quantitative RT-PCR |
| 6981R | GCTTGAGCTTCATGTCGGCG | Semi-quantitative RT-PCR |
| 7057F | GTCACCGCCCACTTCTTCG | Semi-quantitative RT-PCR |
| 7057R | CCTTGAGGAAGGACACCTGC | Semi-quantitative RT-PCR |
| 7058F | CGGCGGGACCGAGAAGTCC | Semi-quantitative RT-PCR |
| 7058R | CCGTACTTGTGCTCGACGG | Semi-quantitative RT-PCR |
| 7063F | TCGTTGTCCGTCGAATCCCG | Semi-quantitative RT-PCR |
| 7063R | CAGGCCTAAGCCCCGGTACG | Semi-quantitative RT-PCR |
| 7098F | GCTCGTACCAGCTCTCCGC | Semi-quantitative RT-PCR |
| 7098R | GGATCGTGTTCTGCGGGTAG | Semi-quantitative RT-PCR |
| 7139F | CGCCCGGAACGGAAAGAACC | Semi-quantitative RT-PCR |
| 7139R | AGTCCTGGTTGGACAGGACG | Semi-quantitative RT-PCR |
| 7154F | CTTCACCTGGTCGCTGTACG  TTCGATCCTCAGGTCGGGGG | Semi-quantitative RT-PCR |
| 7154R | TTCGATCCTCAGGTCGGGGG | Semi-quantitative RT-PCR |
