# Supplementary material for: Streptomyces Volatile Compounds Influence Exploration and Microbial Community Dynamics by Altering Iron Availability
Source: mBio. 2019 Mar 5;10(2):e00171-19. doi: 10.1128/mBio.00171-19 (PMC6401478; doi:10.1128/mBio.00171-19)
Supplement: TABLE S3 [file mBio.00171-19-st003.docx]

**Table S3. *S. venezuelae* siderophore production and uptake genes**

| **Genes** |  |
| --- | --- |
| **Synthesis clusters** | **Predicted siderophore** |
| *sven_0503-17* | Thiazostatin |
| *sven_2566-77* | Desferrioxamine |
| *sven_5413-26* | Unknown siderophore |
| *sven_5472-82* | Rhizobactin-like cyptic siderophore |
| *sven_7032-80* | Coelichelin |
|  |  |
| **Uptake systems** | **Annotation** |
| ***bldK* homologs** |  |
| *sven_4759-63* |  |
| *sven_5150-54* |  |
| *sven_4765-69* |  |
| *sven_4820-23* |  |
| *sven_5369-73* |  |
| *sven_6981-85* |  |
| *sven_7098-02* |  |
| *sven_7135-39* |  |
| *sven_7154-58* |  |
| ***other*** |  |
| *sven_0776-78* | Salmycin transporter CdtABC |
| *sven_0164-66* | Putative siderophore transporter system |
| *sven_1997* | Putative iron-siderophore uptake system |
| *sven_1954-57* | Ferrous iron transport system EfeUOB |
